# Hôpital Des Enfans

**Published:** 1831-04

**Authors:** 


### HÔPITAL DES ENFANS.

### *Softening of the Brain, confined to the Right Optic Thalamus, with Paralysis of the whole of the Left Side of the Body.*

A girl, ten years of age, of a very weak constitution, had been subject for many months to occasional headach. On the 10th of October, she was suddenly attacked, without any known cause, with giddiness, intense pain in the head, and complete immobility of the whole of the left side of the body. She was admitted the following day, under M. Guersent. Her skin was now of a yellowish colour; extreme paleness of the face, which was drawn to the right side; she had an appearance of great depression, and the motions of the face, and of the upper and lower extremities of the left side, were completely paralysed. The sensibility of these parts did not appear to be altered. Intense headach; intellectual faculties clear; she replied to questions, slowly but correctly; her respiration was suspirious, and she had a slight cough; tongue dry; bowels rather costive; pupils largely dilated; pulse quick and full. Twelve leeches were applied behind the ears; nine grains of calomel were given in three doses; purgative enemata, and a low diet.

After various changes in the accessory symptoms, during the following days, to relieve which, cupping, blisters, sinapisms, &c. were had recourse to, the paralysed limbs remained in the same state.

October 29th. The paralysed arm had become œdematous; general weakness much increased; countenance greatly altered.

In the morning of the 2d of November, an abundant hemorrhage took place from the blistered surfaces on the legs, and there was also a slight oozing of blood from the surface of the lips. The little patient was now extremely reduced, and uttered continued complaints. The hemorrhage was suspended by styptics and compression.

On the following night, she accidentally struck her head against the bed, and slightly wounded her forehead: it was with difficulty, however, that the effusion of blood was checked, and two hours afterwards she expired.

*Dissection, forty-two hours after death.* The brain was carefully examined: slight adhesions were found between the choroid plexus and the optic thalamus on the right side; the whole of the right optic thalamus was softened, and reduced to a sort of pap, of the colour of "*café au lait:*" in the centre was found two small masses, of a firmer consistence, and of a grayish colour, about the size of a pea, having the appearance of softened tubercles. The softening extended to the tuberculæ quadrigemini, following the track of the optic nerves. The other parts of the brain were healthy in appearance. The spinal marrow was rather soft. No particular lesion was detected in any other organ.

This case may be opposed to the opinion of those who maintain that paralysis of the inferior extremities depends upon disease of the corpora striata.

